# Intellectual Disability and Assistive Technology: Opening the GATE Wider

**DOI:** 10.3389/fpubh.2017.00010

**Published:** 2017-02-22

**Authors:** Fleur Heleen Boot, John Dinsmore, Chapal Khasnabis, Malcolm MacLachlan

**Affiliations:** ^1^Centre for Global Health and School of Psychology, Trinity College Dublin, Dublin, Ireland; ^2^Centre for Practice and Healthcare Innovation, Trinity College Dublin, Dublin, Ireland; ^3^GATE Group, Essential Medicines & Health Products, World Health Organization, Geneva, Switzerland; ^4^Centre for Rehabilitation Studies, Stellenbosch University, Stellenbosch, South Africa; ^5^Olomouc University Social Health Institute, Palacky University, Olomouc, Czech Republic

**Keywords:** intellectual disabilities, assistive technology, assistive devices, global health, public health policy, health inequality, World Health Organization

## Abstract

The World Health Organization has launched a program to promote Global Cooperation on Assistive Technology (GATE). The objective of the GATE program is to improve access to high quality, affordable assistive technology for people with varying disabilities, diseases, and age-related conditions. As a first step, GATE has developed the assistive products list, a list of priority assistive products based on addressing the greatest need at population level. A specific group of people who can benefit from user appropriate assistive technology are people with intellectual disabilities. However, the use of assistive products by people with intellectual disabilities is a neglected area of research and practice, and offers considerable opportunities for the advancement of population health and the realization of basic human rights. It is unknown how many people with intellectual disabilities globally have access to appropriate assistive products and which factors influence their access. We call for a much greater focus on people with intellectual disabilities within the GATE program. We present a framework for understanding the complex interaction between intellectual disability, health and wellbeing, and assistive technology.

Only 10% of the people who are in need of assistive products actually have access to them, despite such access being claimed to be a human right (1, 2). An assistive product is any product (including devices, equipment, instruments, and software), either specially designed and produced or generally available, whose primary purpose is to maintain or improve an individual’s functioning and independence and thereby promote their wellbeing (3). Common examples of assistive products are spectacles, hearing aids, wheelchairs, prosthetics, communication boards, incontinence products, pill organizers, and therapeutic footwear. Assistive products can improve the quality of life for people with impairments, including the extent of their inclusion and participation in society. However, the use of assistive products by people with an intellectual disability (ID) is a neglected area of research and practice and offers considerable opportunities for the advancement of population health and the realization of basic human rights. About 1% of the total population have ID, with higher prevalence rates in low- and middle income countries (4). ID is defined by the American Association on Intellectual and Developmental Disabilities, the Diagnostic and Statistical Manual of Mental Disorders V, and the International Classifications of Diseases 10 (mental retardation) as an IQ below 70, manifested during the developmental period (<18 years of age), with impairments in adaptive functioning, such as communication skills, social skills, personal independence, school, or work functioning (5–7).

The World Health Organization has launched a program to promote Global Cooperation on Assistive Technology (GATE) to implement those parts of the United Nations Convention on the Rights of Persons with Disabilities referring to assistive technology (3, 8, 9). The GATE program’s objective is to improve access to high quality, affordable assistive products for people with varying disabilities, diseases, and age-related conditions. As a first step, GATE has developed the assistive products list (APL) of priority assistive products to address the greatest needs at population level (10). To be effective, the APL will require countries to develop national assistive technology policies; source appropriate products; train specialized personnel; and develop effective and efficient systems of provision (10).

However, barriers that people with ID experience regarding access to assistive products have not yet been sufficiently considered. Worldwide, people with ID are still generally regarded as a devalued and stigmatized group, and at least part of their relatively poor health status is due to health inequities. People with ID are still often disadvantaged when attempting to access or secure health services and assistive products (11, 12). It is unknown what proportion of people with ID globally actually has access to appropriate assistive products. It has been suggested that for people with ID there is a high rate of underdiagnosis and misdiagnosis; so that too often they do not receive the correct treatment and that the need for rehabilitation arises as a result of absent or ineffective health care (13). The atypical presentation of symptoms by people with ID is often a challenge for their care system. With accurate assessment and appropriate interventions, the use of assistive products can be not only enabling and empowering, but also transformative in facilitating new life skills and opportunities for people with ID.

Compared to the general population, people with ID have a higher prevalence of comorbidities which could be better managed with assistive products (see Figure 1). For instance, motor disabilities are present in a significant proportion (26%) of people with ID (14). Visual impairment has a prevalence of 19.2% in adults with ID compared to 1.9% in adults of the general population. For hearing impairment, the prevalence is 30 vs 17%, respectively; and for dementia, it is 13.1 vs 5.4%, respectively (15). People with ID are now recognized as a group with a disproportionately greater need for assistive products due to higher rates of frailty and multimorbidity (including increased severity and earlier onset) than the general population (16, 17). The result is a greater prevalence of disabilities in daily functioning and mobility with increased care needs and support required (18–20). Multimorbidity (the presence of two or more chronic conditions) is of particular concern with an 80% prevalence rate in adults >50 years with ID (17). Besides the association with age, multimorbidity, and frailty are also associated with a severe and profound level of ID (16, 17). The life expectancy of people with ID is increasing in line with the general population trends. Therefore, the prevalence of older people with ID is also likely to increase along with the demand for access to assistive products (21).

**Figure 1:**
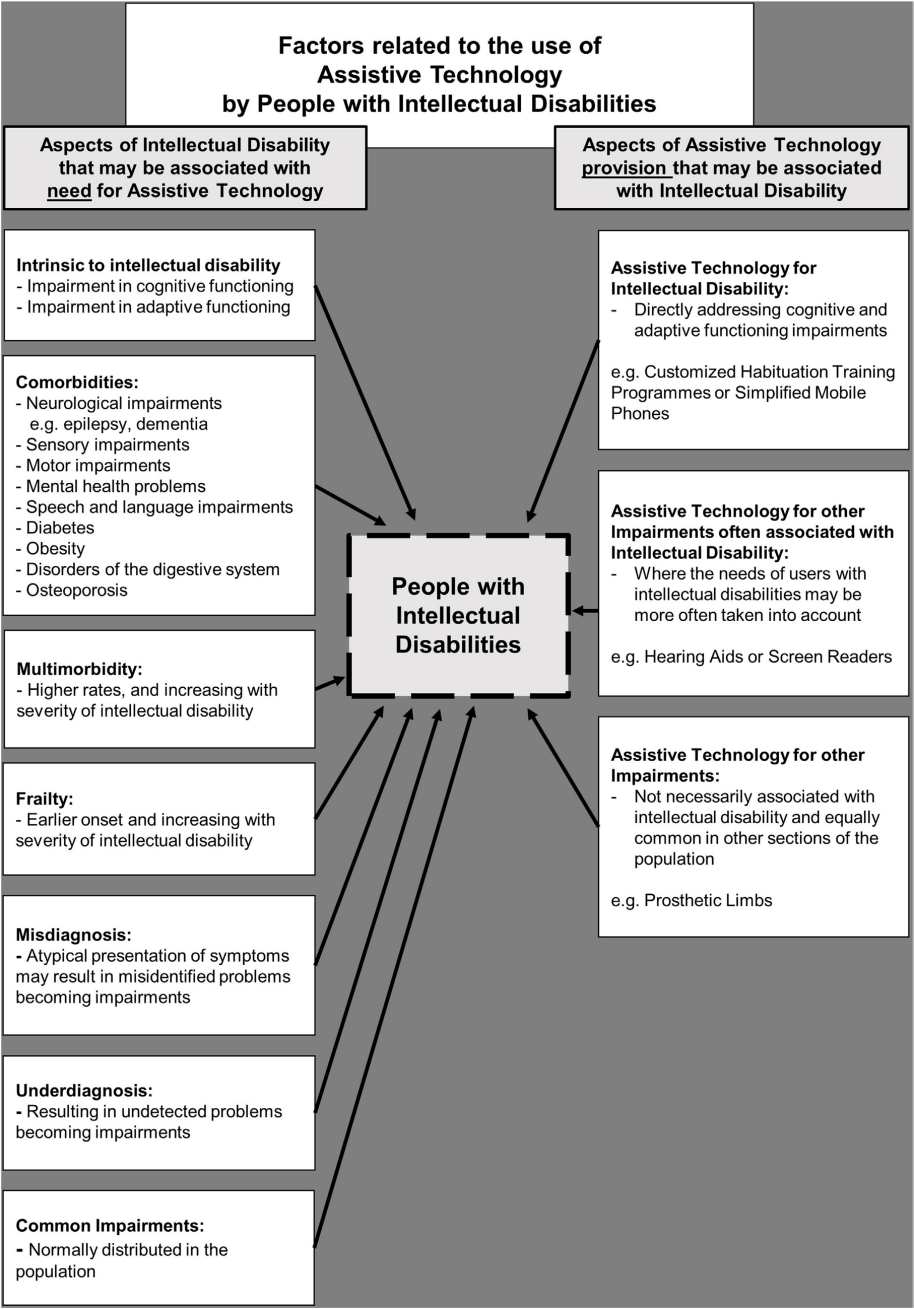
**Factors related to the use of assistive technology by people with intellectual disabilities**.

Access to assistive products presents three distinct challenges if people with ID are going to benefit from the increased provision aspired by GATE (see Figure 1). First, impairments in cognitive and adaptive functioning intrinsic to ID should be adequately catered for within population-level systems of assistive technology policy, products, health care personnel, caregivers, and provision. That means, communication skills and physical examinations by health care personnel need to be adapted to the intellectual and emotional level of the person with ID, to get the correct diagnosis and ensure the appropriate assistive product(s) are prescribed. The use of assistive products requires information, instruction, and support that are both accessible and understandable to the person with ID, if it is to be used effectively. In addition, a multidisciplinary approach to develop protocols for the training and support of people with ID is needed in order to direct the effective use and evaluation of the assistive products. For example, hearing aids require a customized habituation training program adjusted to an individual’s level of ID. This needs to be implemented in collaboration with the speech and language therapist, behaviorist, and caregiver together to help the person with ID to accept and benefit from the use of the new product.

A second challenge for people with ID to benefit from the APL is increased awareness among caregivers and health personnel of comorbidities that people with ID often experience; such as sensory impairments and dementia. These comorbidities may require the use of assistive products, and so the needs of the users with ID must be more often taken into account.

Third, people with ID will experience physical impairments not necessarily associated with ID, which are equally common in other sections of the population. For instance, a person with ID may need to learn to use a prosthesis or walking aids and—as above—the effective use of such products requires information, instruction, and support that is as accessible and understandable as possible. While it is known that the use of assistive products, such as a prosthesis, is influenced by a range of psychosocial factors, such research derives almost exclusively from users of assistive products without ID (22, 23).

Without a concerted and systematic approach to consider the challenges that ID presents, for the users, caregivers, and providers of assistive products, profound inequities in health, in life opportunities, and therefore in the quality of life for people with ID will persist. We call for a much greater focus on people with ID within the GATE program and in particular regarding national initiatives to adopt the APL.

## Author Contributions

FB: substantial contributions to the conception and design of the work; drafting the work; final approval of the version to be published; agreement to be accountable for all aspects of the work in ensuring that questions related to the accuracy or integrity of any part of the work are appropriately investigated and resolved. JD, CK, and MM: substantial contributions to the conception and design of the work; revising the work critically for important intellectual content; final approval of the version to be published; agreement to be accountable for all aspects of the work in ensuring that questions related to the accuracy or integrity of any part of the work are appropriately investigated and resolved.

## Conflict of Interest Statement

The authors alone are responsible for the views expressed in this article and they do not necessarily represent the views, decisions or policies of the institutions with which they are affiliated. None of the authors have any competing interests in the manuscript. The reviewer DB and handling Editor declared their shared affiliation, and the handling Editor states that the process nevertheless met the standards of a fair and objective review.
